# 911 Variability of Categorization of Burn Size in Burn Outcomes Research in a Multicenter Longitudinal Database

**DOI:** 10.1093/jbcr/iraf019.442

**Published:** 2025-04-01

**Authors:** Eloise Stanton, Deja Nicholas, Andrew Humbert, Colleen Ryan, Gretchen Carrougher, Barclay Stewart, Haig Yenikomshian

**Affiliations:** University of Southern California; University of Washington; University of Washington; Shriners Children’s Boston and Massachusetts General Hospital; University of Washington; University of Washington; Keck School of Medicine of University of Southern California

## Abstract

**Introduction:**

Total body surface area burned (TBSA) is the standard metric for comparing burn size. While widely used in acute care for decision-making, its role in predicting long-term outcomes, such as physical recovery and psychological well-being, is less defined. This study systematically analyzed how burn size categorization among reports from a multicenter, longitudinal, patient-reported outcome database is related to long-term outcomes of burn survivors. By analyzing TBSA categorization, we aim to determine if a standardized categorization scheme should be used more widely.

**Methods:**

All publications (1994-2024) from a large multicenter prospective database were included. TBSA data was collected, including stratification methods if used and outcomes analyzed with burn size. Outcomes abstracted include both patient-reported measures (e.g., quality of life, psychological well-being) and objective outcomes (e.g., physical recovery, complication rates). Descriptive statistics were employed to analyze TBSA categorization across studies and their relationship to outcomes.

**Results:**

A total of 106 publications were identified, yielding a combined sample size of 38,660 burn survivors. Of the publications that included data on burn size (n=90, 85), 82 (91%) of manuscripts included TBSA as a continuous covariable and 21 (23%) presented TBSA using categories. The total sample size was 38,660 people living with burn injury. There was a lack of standardization regarding TBSA categories (25 reports). The most common method used was based on deciles (n=6) and quintiles (n=3). The remaining 16 reports had varying TBSA categorizations based on presumed clinically important cut points (e.g., above or below 20% TBSA). About a third of the reports directly assessed how TBSA influenced outcomes, including physical and psychosocial function, return to work, and pain/itch. About a quarter of additional reports included TBSA as a covariable in regression analyses. Of the 32 reports that provided TBSA results, 24 (74.4%) reported findings that TBSA significantly impacted outcomes.

**Conclusions:**

The methods for inclusion of burn size among analyses of patient-reported outcomes are variable. While TBSA is frequently included as a continuous variable, often due to sample size limitations, a minority of reports employed TBSA categories, and cut points vary significantly. This underscores the need for more consistent cut points for TBSA data categories. Doing so would better harmonize studies and allow for more robust analyses of the predictive value of TBSA in long-term burn-survivor outcomes.

**Applicability of Research to Practice:**

This study highlights the need for standardized TBSA categorization to improve the predictive accuracy of long-term outcomes in burn survivors. More research in the broader literature is warranted for a more comprehensive analysis of the impact of %TBSA burn size on outcomes, guiding clinical practice and enhancing burn outcomes research.

**Funding for the Study:**

N/A

